# A human EEG dataset to study cognitive flexibility during auditory discrimination under real-world distractors

**DOI:** 10.1038/s41597-026-07041-5

**Published:** 2026-03-14

**Authors:** Priyanka Ghosh, Kirti Saluja, Arpan Banerjee

**Affiliations:** 1 National Brain Research Centre, NH-8, Manesar, Haryana 122052 India; 2 Present Address: Institute for Cognitive and Brain Health, Northeastern University, Boston, MA 02120 USA

**Keywords:** Attention, Computational neuroscience

## Abstract

Salient sounds in the environment automatically capture our attention, causing a shift of focus away from ongoing goal-directed tasks. Studies of cognitive flexibility can employ such paradigms to examine how the brain reorients attention to the ongoing goal, an ability notably impaired in neurodevelopmental and clinical populations. The current dataset captures attentional reorientation to real-world distractors, featuring 60 naturalistic salient sounds (e.g., ambulance siren, dog bark) presented during goal-directed auditory discrimination tasks involving pure tones, frequency-modulated sweeps, and speech syllables. Novel behavioral and preprocessed electroencephalography (EEG) open-source data are made available from twenty-seven healthy human volunteers performing goal-directed auditory tasks validated across three spectrotemporally different acoustic contexts, along with all task stimuli files. Behavioral data confirmed that distractors significantly modulated task performance across all three auditory tasks, and EEG spectral analyses demonstrated significant power changes linked to auditory distractors. To support accurate source-level analyses, we also provide all individual-specific structural MRIs (3.0 T), 3D head shape digitization files and computed forward models.

## Background and Summary

Navigating complex soundscapes relies on the brain’s ability to prioritize behaviorally relevant auditory information while suppressing irrelevant or distracting sounds^1,2^. In the visual modality, salient distractors are handled via alpha-band modulations driven by the ventral attention network (VAN)^3–5^. Although it is unclear whether similar processes control auditory distraction, the auditory system often relies on visual imagery to make sense of unexpected events (e.g., picturing an ambulance when hearing a siren), suggesting possible cross-modal influences while processing salient sounds. The ability to discriminate between competing sounds is compromised in conditions like tinnitus and autism spectrum disorder (ASD), associated with elevated internal noise and atypical attentional gating, respectively^6,7^. In ASD, an altered excitation–inhibition balance leads to hypersensitivity and abnormal salience attribution, enhancing attention to local acoustic features while impairing the extraction of stable auditory patterns over time^8^. In tinnitus, increased spontaneous activity in auditory pathways introduces internal noise that can mask external sounds, especially near the tinnitus frequency^9^. Even with intact peripheral hearing, individuals with these conditions struggle to segregate sound sources and track speech in noisy environments. Patients with central auditory processing disorder show deficits in binaural integration and spectrotemporal binding, making it challenging to detect rapid acoustic changes, especially in complex acoustic settings^8,10^.

**Fig. 1:**
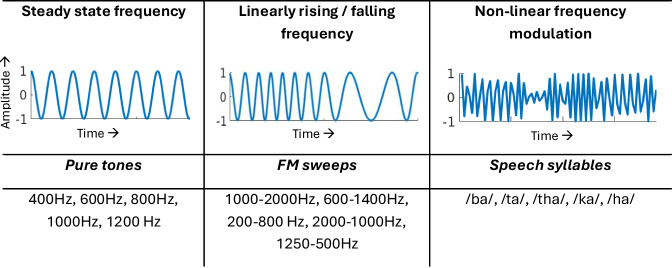
Auditory stimuli conditions. Schematic representation of the three spectrotemporally different auditory contexts and the corresponding stimuli used for the goal-directed auditory tasks.

The existing frameworks in auditory literature suggest that auditory distraction unfolds in three stages: distractor detection, involuntary orienting to the distractor, and voluntary reorienting back to the task^11^. To capture the sub-second neural dynamics underlying the transient stages of attentional reorientation, the present study leveraged the temporal precision of high-density electroencephalography (EEG) in healthy adults performing goal-directed auditory attention tasks. Naturalistic distractor sounds were introduced 200 ms post-onset of goal-directed auditory stimuli^12^ across three acoustic stimulus classes differing in spectrotemporal structure (Fig. 1): (a) pure tones (constant frequency over time), (b) Frequency Modulated (FM) sweeps (increasing/decreasing frequency over time), and (c) speech syllables (non-linear frequency modulations over time). These three classes were chosen based on their distinct acoustic properties and previous evidence underscoring their differential processing in the brain. For instance, non-speech sounds (e.g., pure tones or tonal contours) rely primarily on low-level acoustic feature extraction in primary auditory areas, whereas speech categorization engages higher-order processing beyond the auditory cortex^13,14^. Extant studies further show slightly longer N100m latencies for speech than for tones^15,16^, and vowels eliciting stronger left-hemispheric N100m responses than tones or piano notes^17^. Unlike steady tones, FM sweeps evoke distinct adaptation and enhancement effects in early auditory cortical responses, reflecting specialized processing for dynamic frequency change^18^. By introducing distractors at a controlled time point within each trial across all three tasks, the presented dataset enables the investigation of auditory attention across varying levels of acoustic complexity within a unified experimental framework.

There is a scarcity of EEG datasets that capture the transient neural mechanisms of attention reorientation in the presence of real-world auditory distractors. Bottom-up mechanisms primarily govern the stages of distractor detection and involuntary orienting, while voluntary reorienting to the goal-driven task would involve more top-down control. Our paradigm’s manipulation across the spectrotemporal scale in the presence of common real-world distractor sounds provides a controlled framework for auditory neuroscientists and psycholinguists to systematically study how top-down and bottom-up mechanisms interact during the different stages of auditory distraction^11^. We provide participant-specific forward models to conduct detailed source-level analysis of the scalp-level EEG data. For future reproducibility, the dataset also contains stimulus audio files from all tasks, MATLAB codes to generate the pure tones and FM sweeps, and task-ready presentation codes to run the experiment, which could serve as a benchmark for examining how various sound types interfere with auditory attention in clinical populations with Attention deficit hyperactivity disorder (ADHD) or hearing loss. This dataset can support the development of brain-computer interfaces (BCIs) and modern digital hearing aids that intelligibly mitigate distracting sounds in real time by continuously monitoring auditory patterns in the environment by filtering/amplifying relevant sound signals. The adaptive hearing aids could detect when distractors are degrading auditory processing and dynamically adjust low-level masking centered around the tinnitus frequency. Taken together, the combination of behavioral accuracy, trial-level reaction times, EEG power spectra, and source-level data in our dataset offers avenues to generate behaviorally plausible neurophysiological models of dynamic auditory attention. These models can support the stratification of auditory dysfunction and the design of personalized closed-loop interventions in clinics.

## Methods

### Participants

28 healthy human volunteers (median = 26 years; min = 22 years; max = 36 years; 16 females) participated in this study and self reported no history of neurological or audiological problems. Written informed consent was obtained from all participants for study participation as well as sharing of the de-identified data for research and publication purposes. The study was conducted according to the ethical guidelines and prior approval from the Institutional Human Ethics Committee of the National Brain Research Centre, India (NBRC/2019/007R1). All participants spoke at least two languages fluently, English and Hindi, with a few also speaking a third Indian regional language as their native language. All were at least college graduates and had formal high school training in two languages (English and Hindi/other Indian language). All participants were right-handed and remunerated for their participation. To avoid any attentional bias in the data, participants were discouraged from consuming any stimulant/medication (e.g., tea/coffee/sedatives) within 6 hours before the start of the experiment.

### Experimental design

**Fig. 2:**
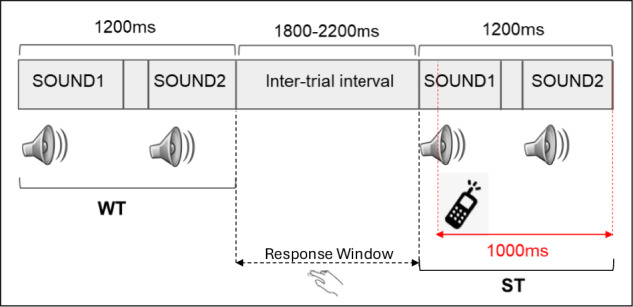
Auditory paradigm. A schematic of the auditory duration discrimination task is shown, which comprises two identical sounds, sound1 and sound2, differing in their individual durations. The goal was to identify the longer/shorter sound as prompted at the beginning of each block. Participants had to respond after the complete presentation of sound2 in each trial, during the inter-trial interval. Each block could comprise tasks with either pure tones (steady-state frequencies), FM sweeps (increasing/decreasing frequencies) or speech syllable sounds (in male/female voice). Within a block, there were 3 categories of trials - Without Saliency (WT), With Saliency (ST) and Neutral Trials (NT), presented in random order. In ST, a salient sound was additionally introduced at 200 ms from the onset of sound1, presented till the offset of sound2.

Participants performed a duration discrimination task using auditory stimuli (Fig. 2). Audio was delivered from a Windows PC (24 bit, 48 kHz) via sound tubes using 10 Ω Neuroscan transducers (Neuroscan, El Paso, TX, USA) coupled to 3 M E-A-RLINK insert earphones (foam tips) for the left and right ear. The system volume setting was held constant across participants and task conditions to ensure that all stimuli were presented within a consistent and comfortable listening range. The task required participants to identify the longer/shorter audio (prompted before the presentation of a block) from a pair of identical sounds differing only in their durations of presentation. There were 6 blocks in total, with 90 trials in each block (Table 1). The target in 50% of the blocks was to identify the longer audio (and shorter audio in the other 50%). All participants used their right hand to respond on the keyboard, pressing the left arrow key if the response was the first audio and the right arrow key if it was the second audio. Each trial was made up of an audio pair (sound1 + delay + sound2) spanning a total duration of 1200 ms (Fig. 2). There was a brief period of silence (delay) between sound1 and sound2 and its length was decided by the individual lengths of sound1 and sound2 such that the total length of a trial was fixed at 1200 ms.

**Table 1:** Trial distribution across conditions.

Trial Information	Tones	Sweeps	Syllables
Total no. of blocks	2	2	2
No. of trials per block	90	90	90
Neutral trials (NT)	30	30	30
Without saliency trials (WT)	30	30	30
Saliency trials (ST)	30	30	30

The audio pair durations used were: 300-500 ms, 350–500 ms and 350–550 ms (and also, 500–300 ms, 500–350 ms and 550–350 ms), standardized after pilot experiments on participants to set easily identifiable perceptual differences between sound1 and sound2. An inter-trial interval jittered between 1800–2200ms was used to minimize the temporal expectancy of the next upcoming trial.

### Auditory tasks

All the participants performed 3 auditory attention-based tasks which consisted of 3 contexts of auditory stimuli, categorized based on their changing frequency characteristics across the temporal scale: ***Tones Task*** – Steady-state frequency, ***Sweeps Task*** – Linearly rising/falling frequency and ***Syllables Task*** – Non-linear frequency modulation (Fig. 1). The *Tones* task stimuli consisted of pure tones of frequencies 400 Hz, 600 Hz, 800 Hz, 1000 Hz and 1200 Hz; the *Sweeps* task stimuli consisted of up and down FM sweeps (1000–2000Hz, 600–1400 Hz, 200–800 Hz, 2000–1000Hz, 1250–500 Hz); and the *Syllables* task stimuli consisted of speech syllables (/ba/, /ta/, /tha/, /ka/, /ha/) in both male and female voices. All the stimuli were matched for loudness and were ramped up/down by 0.5 ms using a cosine-shaped Hanning window at the beginning/end of the sound stimuli to prevent click illusion. The frequency patterns of *Sweep* stimuli were chosen such that they were equi-spaced on a log frequency (Mel) scale. All *Syllable* stimuli were generated using text-to-speech converting software Notevibes (https://notevibes.com/). Sound1 and sound2 of each trial were joined using the audio editing software Audacity (www.audacityteam.org). Each auditory task had three categories of trials called ‘Without Saliency Trials’ ***(WT)***, ‘Saliency Trials’ ***(ST)*** and ‘Neutral Trials’ ***(NT)***, 30 each in every block (Table 1). Within a block, trial presentation order was randomized. The paradigm design in WT was exactly as described above in the experimental design section. In ST, additionally, a salient distractor sound was introduced at a latency of 200 ms from the onset of sound1 which was played till the end of the trial such that post salience duration was 1000 ms long (Fig. 2). The salient distractor sounds ranged from phone bell ring to dog bark sounds which were obtained from Soundbible (http://soundbible.com/), a repository of naturalistic sounds. We used 60 such real-world salient distractor sounds as listed in Table 2. To maintain uniformity, we ensured that ST across all three tasks (Tones/Sweeps/Syllables) consisted of the same distractor sounds and since each task type corresponded to 2 blocks (and each block had 30 saliency trials or ST), none of these sounds were presented more than 3 times to preserve their saliency effect. In NT, sound1 and sound2 were equal in duration (both were either 300 ms, 400 ms, or 500 ms long), but the participants were unaware of this, and hence, NT gave the perception of the most difficult trials. These trials were introduced to keep a check on the participant’s attention, such that if the participant was indeed attending to a task block, it would reflect as increased reaction times in neutral trials over the other two categories of trials.

**Table 2:** List of real-world salient distractor sounds used in the experiment.

LIST OF SALIENT SOUNDS				
Phone ring	Big bell	SMS notification	Fire alarm	Utensils falling
Truck siren	Whistle	Crowd clapping	Thunder	Sheep bloating
Dog Bark	Drum roll	Saw sound	Rooster call	Night crickets
Cat meow	Evil laugh	Car racing	Cycle horn	Sniffing
Child cheering	Steam engine	Bomb drop	Aeroplane	Brooming
Woman screaming	Dog growling	Angry growl	Baby crying	Milk pouring
Welding	Fireworks	Guitar playing	Devotional chanting	Tyrannosaurus
Brook	Flock of seagulls	Birds chirping	Clash of swords	Coughing
Eagle screeching	Bats screeching	Lion roaring	Helicopter	Nose blow
Spanish no. counting	Funny human sound	Child speaking gibberish	Car engine starting	Tongue rolling
Coins dropping	Gibbon monkeys	Bottles rattling	Toilet flush	Generator start
Gunshot	Cartoon	Party crowd	Motorbike	Wind chimes

### EEG data acquisition

All participants were seated in a dark, soundproof room during the experiment and were asked to focus on a central ‘ + ’ fixation displayed on the computer screen placed right in front of them to minimize eye movements. EEG data were collected from 63 active electrode channels using Brain Vision EEG recording system and acquisition software with the reference electrode at FCz and the ground electrode anterior to AFz. The channel impedances were constantly monitored and maintained below 10 kΩ. Data were acquired at a sampling rate of 1000 Hz and a 50 Hz line noise filter was applied online in the Brain Vision software. All the responses were marked by receiving triggers at key presses on a computer keyboard recorded through the Neurobehavioral Systems (NBS) Presentation software. The participants were briefed about the task at the beginning of the experiment, and a short demo of the paradigm was presented to them for a better understanding of the task. They were instructed to listen to the stimuli carefully during the experiment and to respond as fast and as accurately as possible to all the trials. If the participants responded more than once to a trial, only the first response was considered for further analysis. The blocks were randomized across participants, and a rest period was allowed after every block to minimize fatigue. After the EEG session, the 3D location of electrodes were recorded using a Polhemus Fastrak system with a set of fiducial points (Cz, nasion, inion, left and right pre-auricular points) while the EEG cap was placed on the participant’s head.

### EEG data preprocessing

The EEG data were pre-processed using the EEGLAB toolbox (Delorme and Makeig, 2004) and custom-written scripts in MATLAB (www.mathworks.com). The raw data were first imported and bandpass filtered between 1–90 Hz using zero-phase Hamming-windowed sinc FIR filter. The filtered data corresponding to time stamps before and after a block presentation period were removed. The data were visually inspected at this stage and one participant’s data was discarded because there were more than 5 noisy channel recordings. One channel (T8) in another participant was noisy and was hence interpolated to neighboring channels. Independent component analysis (ICA) was then applied block-wise to each participant’s data and the ICs corresponding to eyeblinks and eye movements were visually identified and removed. Next, the data were average re-referenced, epoched and sorted according to the trial categories, i.e., WT, ST and NT, based on their trigger information. Each epoch was 1400 ms long, with 200 ms of pre- and 1200 ms of post-stimulus activity. Each trial (epoch) was baseline corrected using the 200 ms pre-stimulus activity and any linear trends were removed. To ensure that our neural data were free from any muscular or electrocardiograph artifacts, we further set a threshold of ±75 µV such that trials with an amplitude more/less than this threshold were rejected from all the channels.

### Analyses

The reaction time and accuracy of each trial were recorded across all three tasks using NBS Presentation software. Trial-specific accuracies were either coded as ‘hit’ or ‘incorrect’. Missed trials were not recorded. The reaction time was defined as the duration from the onset of a trial (0 ms) till the participant hit the response key. Responses were typically made after the presentation of sound 2, at any point during the inter-trial interval. To have a better estimate of the participant’s engagement with the task, the blocks with accuracy <70% were rejected for neural data analysis. Only one block from a participant (subject #10) in the tones task was thus rejected. Reaction times (RTs) less than 100 ms were treated as anticipatory responses or accidental key presses and were thus excluded^19^. Responses made after the commencement of the subsequent trial, and the trials without any responses were also not considered for analysis. All trials from the remaining 27 participants were sorted based on the above criteria and their reaction times and % accuracies were computed and compared across WT, ST and NT for tones, sweeps and syllables tasks.

## Data Records

All task stimuli used in the experiment, EEG, MRI and behavioral datasets from 27 participants and the *NBS* presentation codes have been shared on Open Science Framework at https://osf.io/zx2up for public use^20^.

## Stimuli

The Stimuli_Tones folder contains .wav audio files named according to the following convention: file names begin with P (pure tones), followed by the tone frequency (e.g., P1000hz_*), and the duration of the two tones in milliseconds, in their presentation order (e.g., *_300_500_*). For files in the ST category, this is further followed by sal (saliency) and the distractor number (1–60). For example, P1000hz_300_500_sal19.wav corresponds to a tone pair of 1000 Hz, where the first tone is 300 ms long and the second tone is 500 ms long, presented along with distractor sound #19. The Stimuli_Syllables folder follows the same convention, with the only difference being that file names begin with the syllable identifier (e.g., /ba/, /ta/, etc.). The Stimuli_Sweeps folder contains dynamic sweep files beginning with the letter D, followed by the start and end frequencies (e.g., _1000_2000hz) and the durations of the two sounds in order of presentation. For NT (neutral) category, file names in all three folders contain ‘neut’ and a single duration value (e.g., *_neut_500), indicating that both sounds had the same duration (e.g., 500 ms each).

### Presentation codes

All the stimuli described above have been incorporated into the stimulus presentation codes shared in the repository in the folder *NBS Codes*. For each task (Tones, Sweeps, and Syllables), two files are provided corresponding to the two experimental blocks. In Tones_block1, Sweeps_block1, and Syllables_block1, participants are instructed at the beginning of the task to identify the longer tone in the duration discrimination task, whereas in Tones_block2, Sweeps_block2, and Syllables_block2, they are instructed to identify the shorter tone. All codes are ready to run as scenario (.sce) files with NBS Presentation Software.

### Behavior

Behavioral data include subject-level reaction times (*_RT_*) and accuracies (*_Accuracy_*). Each condition (Tones/Sweeps/Syllables) was presented in two blocks, with the block number indicated by *_1 or *_2 in the.mat file names. Trial categories are denoted as:

*_WT_* for trials without saliency
*_ST_* for trials with saliency
*_NT_* for neutral trials

### EEG

Preprocessed EEG data for each subject are provided in the folders EEG_Tones, EEG_Sweeps, and EEG_Syllables. Each subject’s.mat file (sub01.mat, sub02.mat, ….sub27.mat) contains three variables: *wt* (without saliency), *st* (with saliency), and *nt* (neutral) representing EEG recorded during the presentation of the respective categories of trials. Each variable is a 3D matrix of form time-series X channels X trials. The time-series are 1400 ms long, including a 200 ms pre-stimulus baseline and 1200 ms of post-stimulus activity. Stimulus onset occurs at 201 ms, and for saliency trials (ST), distractor onset occurs at 401 ms.

## Technical Validation

### Behavioral Performance

**Fig. 3:**
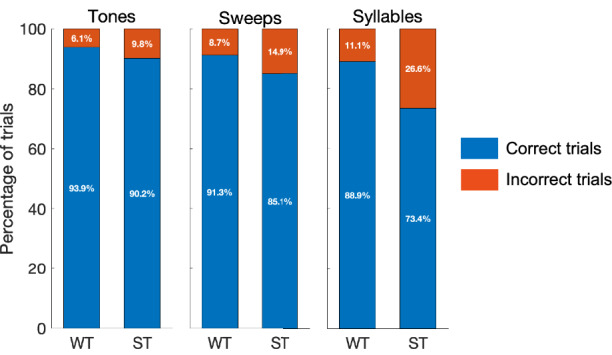
Accuracy across tasks. The stacked barplots represent behavioral accuracies in percentage of correct and incorrect trials from all participants in Without Saliency (WT) and With Saliency (ST) trial categories across Tones, Sweeps, and Syllables Tasks. Overall, the accuracies show a decreasing trend with increasing acoustic complexity from Tones to Syllables.

**Fig. 4:**
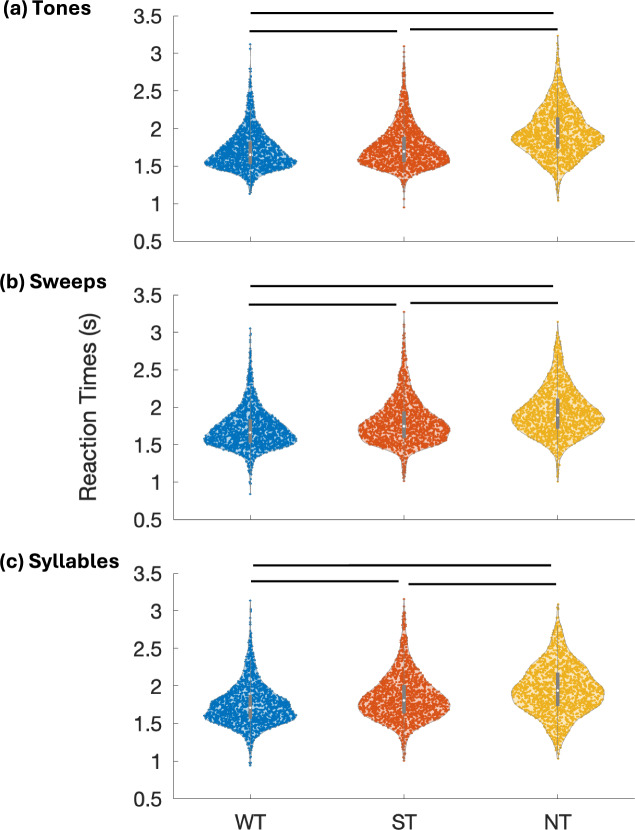
Reaction times (RTs) across tasks. The violin plots represent the distribution of reaction times of all **Without Saliency Trials (WT),** **With Saliency Trials (ST)** and **Neutral Trials (NT)** for (**a**) Tones Task (**b**) Sweeps Task and, (**c**) Syllables Task. The white dot at the center of each violin represents the median of the distribution and the colored dots making the violin are individual reaction times of trials. Significant pairwise differences between trial categories, as determined by Wilcoxon signed-rank tests, are indicated by black horizontal lines (p < 0.0001).

The overall hit responses (% correct responses) of participants in trials without saliency (WT) for Tones, Sweeps and Syllables conditions were 93.9, 91.3 and 88.9, respectively which dropped in the presence of salient distractors (ST) to 90.2, 85.1 and 73.4, respectively **(**Fig. 3**)**. The overall incorrect responses (in %) in trials without saliency for Tones, Sweeps and Syllables conditions were 6.1, 8.7 and 11.1, respectively, which increased to 9.8, 14.9 and 26.6, respectively, in the presence of salient distractors **(**Fig. 3**)**. Since the neutral trials (NT) were not designed to have any correct responses, they were not a part of the accuracy comparisons. The reaction time medians of all trials in WT, ST and NT corresponding to Tones task were 1649.3 ms, 1694.6 ms and 1905.9 ms, respectively; for Sweeps task were 1663.7 ms, 1745.2 ms and 1891.3 ms, respectively; and for Syllables task were 1708.1 ms, 1802.9 ms and 1938ms, respectively (Fig. 4). In all three tasks, the RTs of NT were significantly higher than those of ST (p < 0.0001) and WT (p < 0.0001), whereas the RTs of ST were significantly higher than those of WT (p < 0.0001), as seen using Wilcoxon signed-rank test. The overall behavioral trends reflect a drop in accuracy and an increase in reaction time with increasing acoustic complexity (Tones < Sweeps < Syllables).

### Power spectra

The pre-processed EEG data was Fourier transformed using the mt_spectrumc (multi-taper method) function of Chronux toolbox. As blocks with behavioral performance above 70% accuracy were considered, both correct and incorrect trials were included to have a good signal-to-noise ratio. For all these trials, we used 1200 ms of EEG recording post-stimulus onset across WT, ST and NT categories. The sampling frequency was set to 1000 Hz, time-bandwidth product to 3, Slepian tapers to 5 with padding set to zero (default). Using these parameters, we obtained trial-by-trial power spectra between 1–45 Hz.

**Fig. 5:**
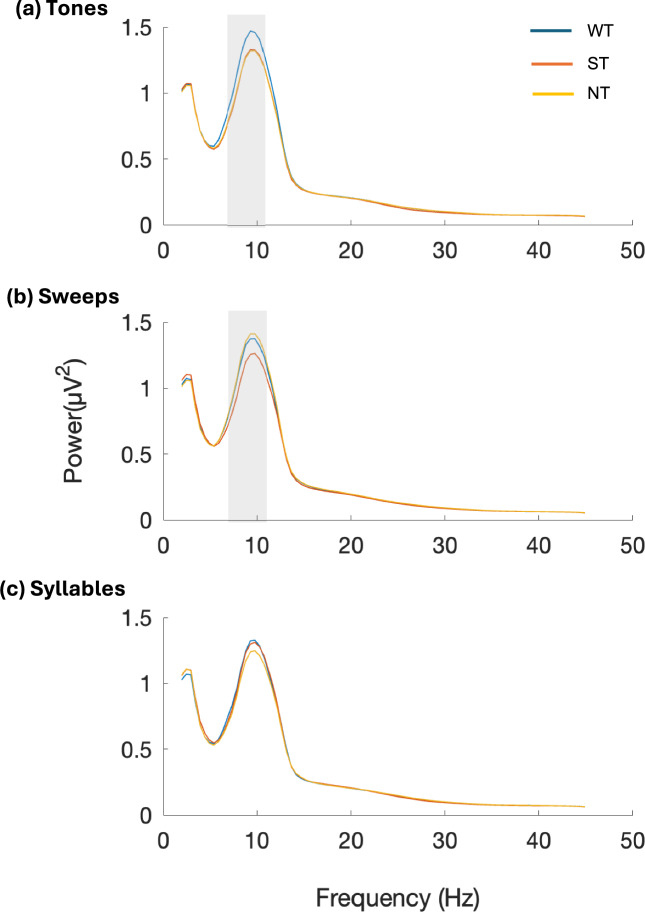
Power spectral density. The mean global power spectra plots corresponding to (**a**) Tones, (**b**) Sweeps and (c) Syllables tasks are shown for without saliency (WT), saliency (ST), and neutral trial (NT) categories. The gray shading in the Tones task represents significantly higher powerspectra of WT as compared to ST and NT at 7–10 Hz. The gray shading in the Sweeps task represents significantly higher powerspectra of WT and NT as compared to ST between 7–10 Hz.

EEG power spectrum was computed for each trial using the 1200 ms post-stimulus window. These spectra were first averaged across trials within each condition, and then collapsed across all 63 electrodes to yield grand-average power spectra for the WT, ST, and NT conditions across the Tones, Sweeps, and Syllables tasks. (Fig. 5). Trial-level power spectra comparisons across WT, ST and NT categories were computed for all frequency bands using Student’s t-test. The power spectra of the trials without saliency (WT) were consistently higher than the power spectra of trials with saliency (ST) in the Tones (p = 0.014), Sweeps (p = 0.028) and Syllables (n.s.) tasks between 7–10 Hz (alpha frequency band). In the Tones task, WT is also significantly higher (p = 0.015) than NT, whereas NT is significantly higher (p = 0.006) than ST in the Sweeps task between 7–10 Hz.

## Usage Notes

The 3D location of electrodes of each participant is provided in .DAT format in the folder *3D_locs*. The T1-weighted structural MRI images (MPRAGE) of all 27 participants were collected from a Philips Achieva 3.0 T MRI scanner using the following acquisition parameters: TR = 8.4 ms, FOV = 250 × 230 × 170, flip angle = 8°, and fiducials marked at nasion, left and right pre-auricular points with Vitamin E capsules. The MRI files are provided in NIfTI (.nii) format in the folder *MRI*. Latest version of SPM software package can be used to read the MRI files for implementing further source localization methods in the dataset.

The presented EEG dataset offers an extensive resource to test neural signatures of auditory distraction and oscillatory control within attentional networks like the ventral attention network, to determine if they generalize across stimulus classes, and to map how these dynamics shape behavior during real-world distracting sounds. Previous studies have consistently shown that more alpha power is related to better performance, for example, faster reaction times reflect a weaker interference by the distractor^21–23^. The current behavioral dataset provides block-wise trial-specific reaction times and accuracies of all participants, offering a strong test case to understand (a) how reaction times and accuracies relate to an individual’s alpha power associated with processing distractor sounds and (b) behavioral performances as a function of spectrotemporally varying acoustic contexts.

## Data Availability

The dataset is available at https://osf.io/zx2up.

## Code availability

Codes used for the generation of accuracy, reaction times and power spectrum plots can be found in the *Matlab Codes* folder of https://osf.io/zx2up.
